# dbCerEx: A Web-Based Database for the Analysis of Cervical Cancer Transcriptomes

**DOI:** 10.1371/journal.pone.0099834

**Published:** 2014-06-11

**Authors:** Limin Zhou, Wei Zheng, Majing Luo, Jing Feng, Zhichun Jin, Yan Wang, Dunlan Zhang, Qiongxiu Tang, Yan He

**Affiliations:** 1 Hubei Maternal and Child Health Hospital, Wuhan, Hubei, P.R. China; 2 Yichang Humanwell Pharmaceutical Co.,Ltd, Yichang, Hubei, P.R. China; 3 College of Fisheries, Huazhong Agricultural University, Wuhan, P.R. China; 4 College of Life Sciences, Wuhan University, Wuhan, P.R. China; 5 International school of software, Wuhan University, Wuhan, P.R. China; Georgia Institute of Technology, United States of America

## Abstract

**Background:**

Cervical cancers are ranked the second-most hazardous ailments among women worldwide. In the past two decades, microarray technologies have been applied to study genes involved in malignancy progress. However, in most of the published microarray studies, only a few genes were reported leaving rather a large amount of data unused. Also, RNA-Seq data has become more standard for transcriptome analysis and is widely applied in cancer studies. There is a growing demand for a tool to help the experimental researchers who are keen to explore cervical cancer gene therapy, but lack computer expertise to access and analyze the high throughput gene expression data.

**Description:**

The dbCerEx database is designed to retrieve and process gene expression data from cervical cancer samples. It includes the genome wide expression profiles of cervical cancer samples, as well as a web utility to cluster genes with similar expression patterns. This feature will help researchers conduct further research to uncover novel gene functions.

**Conclusion:**

The dbCerEx database is freely available for non-commercial use at http://128.135.207.10/dbCerEx/, and will be updated and integrated with more features as needed.

## Introduction

Cervical cancers account for the second-most gynecological cancer death cases worldwide, and this situation is worse in developing countries due to the lack of adequate organized screening programs. It is believed that Human Papilloma Virus (HPV) infections are the major causes of invasive cervical cancer [1].

Whole- genome expression profiling has revolutionized in the way we study disease and basic biology. Since 1997, the number of published results based on an analysis of gene expression microarray data has grown from 30 to over 5,000 publications per year [2]. DNA microarray technologies aim at simultaneous measurements of the expression of thousands of genes in one single experiment. Over the past few years, this technology has facilitated better understanding of the complex and heterogeneous molecular characteristics of cancers and helped to improve treatment in cancers. For example, HOXC10 gene at first was identified to belong to the 171 significantly up-regulated genes in the cervical squamous cell carcinomas (SCC) relative to normal cervix samples from DNA microarray, which was later identified as a key mediator of invasion in cervical cancer [3]. Archival RNA samples of 25 patients were hybridized to Stanford microarray chips to build a seven gene scoring system [4]. This gene expression pattern could help to identify patients with cervical cancer who can be treated with radiotherapy alone. The specific expression profiles of candidate genes were selected to identify historical subtypes of cervical cancer [5]. Furthermore, numerous candidate biomarkers and therapeutic targets have been identified in other cancers.

However, for most of the published microarray studies, only subsets of genes have been reported to demonstrate the authors’ hypothesis. The complete microarray datasets are stored in an unsystematic manner, and useful only to those with computational expertise. Also, RNA-Seq data has become more standard for transcriptome analysis and is widely applied in cancer studies. While for most of the experimental researchers, there also remain difficulties to utilize these cancer microarray databases and RNA-Seq data to solve biological questions. For example, if one novel gene of interest has a correlated (positive or negative) expression pattern with an apoptosis-related gene, it indicates that they may share the same regulatory mechanism, which could provide the potential research proposal for the novel gene.

Here we present dbCerEx, a database of gene expression profiles generated from DNA microarray experiments and RNA-Seq data. The database is provided with an integrated web-based utility, which has made the data easily accessible to the cervical cancer research community. According to this method, the experimental researchers could identify novel cervical cancer related genes and explore the relationships among them.

## Construction and Content

### Microarray and RNA-Seq Data

The microarray expression data (GSE matrix files) and platform annotation (GPL files) were retrieved from Gene Expression Omnibus (GEO) database [6] via a R [7]/Bioconductor [8] ‘GEOquery’ package [9]. The RNA-Seq data were retrieved from The Cancer Genome Atlas (TCGA) Data Portal [10], which contains clinical information, genomic characterization data and high level sequence analysis of the tumor genomes. The data was then log (base 2) transformed and median centred. To avoid computational error during calculation, the row that contained ‘NA’ value would be omitted.

The experiments were processed via various platforms (Table 1). To make the expression data searchable regardless of the platforms, the probes were remapped to official gene symbols. However, instead of gene symbol assignment information, some GPL files provided only NCBI GenBank [11] or NCBI Refseq [12] Accession Numbers mapping to probes. To solve this problem, the ‘gene2refseq’ and ‘gene2accesion’ files were retrieved from the NCBI ftp server via ftp://ftp.ncbi.nlm.nih.gov/gene/DATA/. A Perl script was used to map gene symbols to these GenBank or RefSeq Accession Numbers, and eventually to the microarray probes. The gene expression flat files were stored for later accessing.

**Table 1:** List of GEO accession number, published year and expression platforms of microarray experiments and RNA-Seq data used in this study.

	GEO Acc.*	Year	Expression Platform	Sample Information	Reference
1	GSE5787	2006	Affymetrix HumanGenome U133 Plus2.0 Array	Sixty-six flash-frozen punch biopsies wereobtained from 16 patients with cervical cancer.	[13]
2	GSE3578	2007	GE Healthcare/AmershamBiosciencesCodeLink HumanWholeGenome Bioarray	Twenty-eight squamous cell carcinoma ofcervix from 24 patients were taken as biopsy sample before treatment and during treatment	[14]
3	GSE6791	2007	Affymetrix HumanGenome U133Plus 2.0 Array	84 cervical cancers, head and neck cancersand site-matched normal epithelialsamples from 20 patients	[15]
4	GSE10372	2008	SentrixHuman-6 ExpressionBeadChip	32 snap-frozen tissues from 68 cervical carcinomaspatients who underwent radical hysterectomywith bilateral lymphadenectomy between 1991 and 2005.	[16]
5	GSE9750	2008	Affymetrix HumanGenome U133A Array	A total of 66 samples were included, which include 33primary tumors, 9 cell lines, and 24 normal cervicalepithelium.	[17]
6	GSE20167	2010	GE Healthcare/AmershamBioscienceCodeLink HumanWhole GenomeBioarray	A total of 80 cevical cancer samples of following histology were included in this study: 54 squamous cellcarcinoma, 18 adenosquamous carcinomas, 6 adenocarcinoma,and 2 others	[5]
7	GSE29570	2012	Affymetrix HumanGene 1.0 ST Array[transcript (gene)version]	The polymorphism of mtDNA D-Loop was investigatedin 187 cervical cancer patients and 270healthy controls.	
8	GSE39001	2013	Affymetrix HumanHG-FocusTarget Array	43 HPV16-positive cevical cancer and 12 healthy cervical epitheliums using the HG-Focus microarray	
9	GSE27469	2013	Illumina HumanWG-6 v3.0Expressionbeadchip	82 patients with cervical cancer, stage 1b bulkythrough 4a, were included	[18]
10	TCGA-CESC	2014	RNASeqTCGA	The total number of Cervical squamous cellcarcinoma and endocervical adenocarcinomasamples is 190.	[10]

*NCBI Gene Expression Omnibus Accession number, it can be used to retrieve the microarray experiment data via http://www.ncbi.nlm.nih.gov/geo/.

### Predefined Gene Set

One important feature of this database is that it enables users to search similar gene candidates with genes they are studying based on the expression patterns. Relying on this method, researchers may find mechanisms among these genes, which may become a promising approach to discovering novel gene function. The gene sets predefined in the databases were retrieved from various sources and divided into two main categories: Gene Ontology (GO) [19] and Pathway. As shown in Table 2, the GO set consists of Biological process, Molecular functions and Cellular Component. While the Pathway set consists of KEGG [20], BIOCARTA (www.biocarta.com) and REACTOME [21]. Human species of the gene sets were used in this work.

**Table 2:** Predefined Gene Sets.

Category	Gene set title	Number of gene sets
**Pathway**	BIOCARTA	217
	KEGG	186
	REACTOME	674
**Gene Ontology**	Biological process	825
	Cellular component	233
	Molecular function	396

### Gene Expression Cluster Analysis

The unsupervised hierarchical clustering algorithm was introduced to find the similar genes based on expression patterns. This attempt was processed using a combination of distance metrics and linkages. In this study, the distance from gene x to gene y defined as 1-r_xy_, where r_xy_ represents the Pearson Correlation of gene x and y:
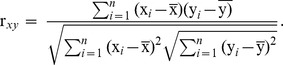



### Database Implementation

The dbCerEx database is a web-based utility combining a MySQL (http://www.mysql.com/) database management system [MySQL 5.5.32 (Community Server) with InnoDB engine]. The front-end web interface is enhanced by a java script framework, Bootstrap 2.3.1 (http://getbootstrap.com/). The PHP [version 5.3.10] (http://www.php.net/) applications receive the query from the user, are connected to the database to gather data, call external Perl and R scripts to process statistical analyze and generate HTML pages displaying results.

## Utility and Discussion

The dbCerEx database is provided by a web-based interface. Users can start the search by entering one interested gene in the top input box, and then click on ‘Search’ button. A gene list will be shown in a new page for all the genes related to input gene keyword. Users can select a gene from the list according to the description to do expression analysis.

By clicking a gene, a general summary including full name, aliases and external links such as HNGC, Entrez Gene, Ensembl. MIM and Genecard for this gene will be shown. In the same page, users are allowed to set the parameters of expression analysis in cervical cancer. Users can enter an interested gene set by hand or from the gene set list such as KEGG, BIOCARTA, REACTOME and Gene Ontology. Users can select dataset from the precompiled cervical cancer expression datasets from microarray and RNASeq, or just provide a GEO accession number. By clicking the Submit Query button, the samples for the selected dataset will be listed. Users can select all or some interested samples to do expression analysis.

**Figure 1:**
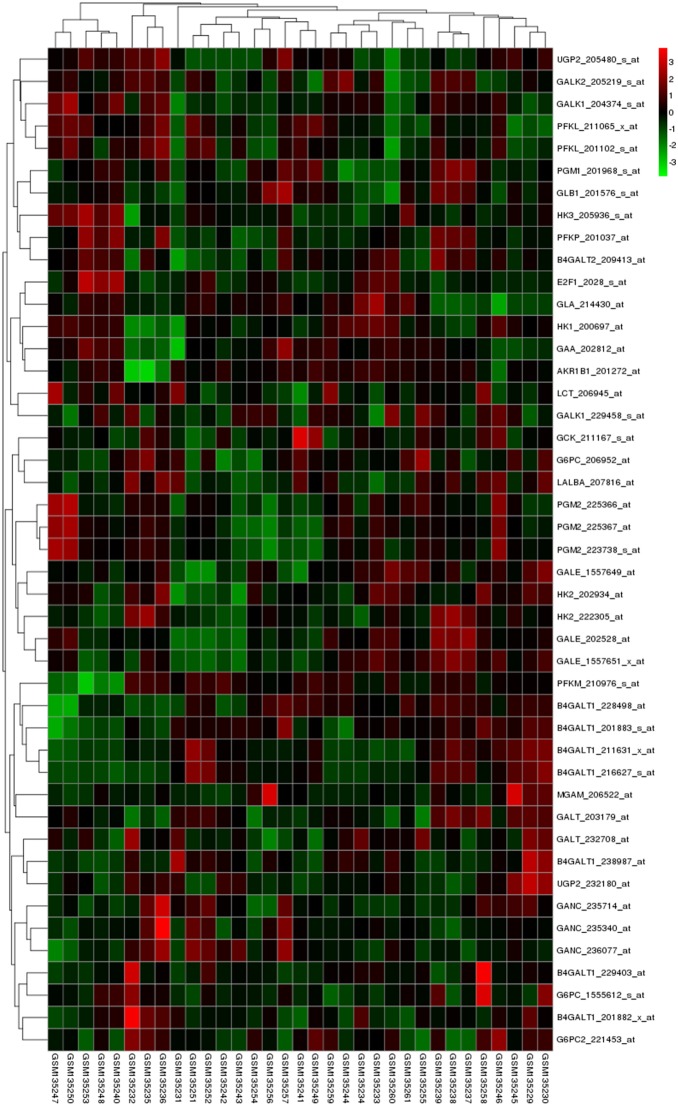
A heatmap showing the hierarchical clustering of the interested gene and geneset.

**Figure 2:**
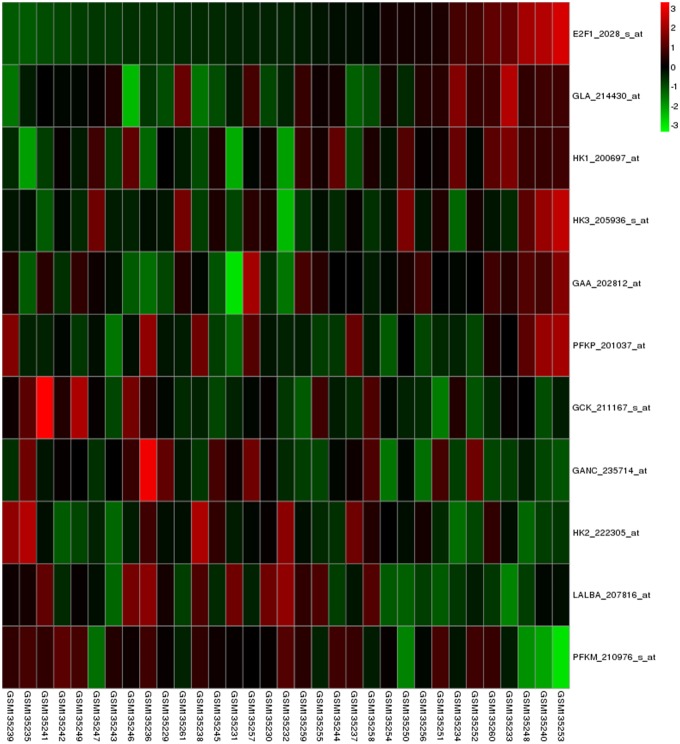
A heatmap showing the genes that are positively or negatively correlated with the interested gene. The genes that have significant pearson correlation with the interested gene were selected to plot a heatmap. The samplers are in the column, and ordered by the expression of the interested gene.

A heatmap displaying the hierarchical clustering of genes and samples will be shown (Figure 1). In addition, a heatmap that includes the significantly positively or negatively correlated genes with the interested gene will be also offered (Figure 2). The pearson correlation and p value will be shown as a table at the right side of the heatmap.

## Conclusion

We present dbCerEx, a database containing cervical cancer gene expression profiles. In addition, it provides a novel utility for gene expression similarity search within certain interested gene sets. It is believed that dbCerEx is a powerful platform for bioinformatics discovery that brings cervical cancer microarray data and RNA-Seq data, and analysis of the cervical cancer research community with easy reach.

## Availability and Requirements

The dbCerEx database website is available free of charge as a web application at: http://128.135.207.10/dbCerEx/.
